# A Case from My Note Book

**Published:** 1854-05

**Authors:** J. C. Hinsey

**Affiliations:** Pekin, Illinois


*A Case from my Note Book.* By J. C. Hinsey, M.D., Pekin. Illinois.

John Ryan, aged 22 years, large and muscular, received an injury in the back from a brick bat during a riot between six or seven Irishmen, Sunday, August 14th, about 4 o'clock P. M.

On arriving at the place where the fight occurred, in company with a number of citizens, the countenance of the patient attracted my attention immediately, as betraying evidence of severe injury. I expressed my opinion to that effect to the men who seemed to have the patient in charge, (his comrades), and who were trying to make him sit up in a buggy for the purpose of driving home, and to escape the officers of the law. The patient being in a state of syncope, of course they could do nothing with him. I voluntarily directed him to be removed from the buggy, and laid in a horizontal position. Thinking that the syncope would soon pass off, and not having anything better, I prescribed a little tinct. camph. internally, and to his face; but the circulation and respiration not returning, I proceeded to examine his body for the marks of the violence. I found a small contusion with ecchymosis on the third, fourth and fifth false ribs on the left side, immediately in front of the angle. The countenance of the patient became cadaverous, respiration performed at long intervals, with no pulse in the radial artery and very feeble action of the heart. Face and hands covered with cold clammy sweat. Extremitics ice-cold with no sensation of touch.

Had patient removed to a bed, ordered brandy *internally*, *hot dry bricks around his feet and legs.* 10 o'clock P. M., reaction fully re-established, arterial action disturbed; pulse 129, rather feeble; complains of great pain in the the bowels, especially in left lumbar region; tenderness of pressure; respiration hurried and difficult; sense of suffocation, thirst great, with occasional vomiting; same anxious expression of countenance, as generally attends severe uterine hemorrhage. Not feeling satisfied that the state of the pulse and cther symptoms would warrant bleeding, I ordered the following cathartic:

℞ Sub. Mur. Hydr., grs x,

Pulv. Jallappæ, " v, M.

15th.—8 o'clock A. M. Cathartic of last night not operated; great pain in the bowels, increased by pressure; pulse 140, *full* and *strong;* eyes a little suffused; respiration 56 per minute, laborious breathing, principally with the abdominal muscels; passed some urine during night, in small quantities and with great difficulty.

Took 32 oz. blood from the right arm, and ordered the following: ℞ Ol. Ricini ℥j.

Ol. Terebinth. ʒji. M.

One o'clock P. M. Oil operated twice, which gave some relief for a short time. Patient about the same as at morning visit; pulse 140, not very strong; respiration 50 per minute and laborious; pain continues in the bowels; great thirst with ejection of the water; tongue becoming a little red and dry; skin hot and dry. Gave ℞ Sub. Mur. Hydr., grs. iv.
Ipecac, " jii.
Nit. Potass., " v. M. every three hours.

Five o'clock, P. M. Dr. Hoffman called in to counsel—Diagnosed inflammation of the lungs, stomach and bowels Patient nearly the same as at last visit. Pulse 140, full and quick; respiration 50 per minute; great anxiety of countenance.

Gave ℞ Ext. Aconitum, grs. x,
Aqua. ℥j. M. every two hours a teaspoon full.

Nine o,clock, P. M. Saw the patient with Dr. J. S. Maus. Is some worse; pulse 145 full and strong; respiration the same as last noticed; skin hot and dry; expression more anxious; pain in bowels still continues. Bled again 32 oz. from arm—bleeding gave great relief—continue cal. powders every 4 hours with small pieces of ice to swallow.

16th.—Nine o'clock, A. M. Patient sinking, with cadaverous countenance; pulse flickering irregular; constant vomiting light yellow bilious matter; skin yellow with inject on of bile, also passing billious matters from the bowels every few minutes; some degree of suffocation; has not retained any of the medicine since last night; extremities cold. He expired at half past 9 o'clock.

Autopsy, seven hours after death:—Present, Drs. Hoffman, Wright and Maus.

General appearance.—Ecchymosis of whole posterior surface of the body; skin deeply tinged with yellow colored matter of the bile. The small contusion on ribs before mentioned; opened the thoracic cavity by carrying incision from the superior extremity of the sternum to the umbilicus, and dividing the ribs at their cartilaginous junction after dissecting back the pectoral muscles. Lungs appeared to be forced towards apex of the chest. Engorgement of the upper lobe of lung; the posterior surfaee of the upper lobe of left side shows evident marks of recent inflammation. Base of the lower lobe also in state of inflammation, being greatly engorged with blood.

*Heart and Large Vessels.*—The external appearance of the heart is natural, both in size and color. Both ventricles empty; the valves normal; vessels in natural condition.

*Abdomen.*—Small intestines much stained with blood of a greenish color; omentum much lacerated and also stained with blood; found about one gallon of blood in the cavity, principally on the left side and having evidently come from the splenic vessels. That organ is nearly three times as large as natural, and engorged with very dark green blood; the vessels of the spleen were in such a condition that we could not determine exactly the point of the hemorrhage. The liver was somewhat enlarged, apparently from previous disease. The stomach, like all the rest of the organs in the abdominal cavity, was stained with blood; mucus membrane of stomach healthy; about half a pint of vitiated bile in that cavity.

The immediate cause of dissolution having been discovered, and time not permitting, the examination was not carried any further.

I remark that the patient had been subject to repeated and obstinate intermittent attacks, and suggest that the abnormal size and softening of the substance of the spleen, would render it liable to such an accident as the above. When we reflect that death is often produced by such slight causes, we ought to be reminded not to treat with too much indifference those apparently trifling cases; for they may become the most serious. It is true, perhaps, that such cases will not happen except in subjects rendered peculiary liable to them by previous disease, but of such the practitioner cannot always judge before hand.

